# Ranking Cognitive Flexibility in a Group Setting of Rhesus Monkeys with a Set-Shifting Procedure

**DOI:** 10.3389/fnbeh.2017.00055

**Published:** 2017-03-23

**Authors:** Tatiana A. Shnitko, Daicia C. Allen, Steven W. Gonzales, Nicole A. R. Walter, Kathleen A. Grant

**Affiliations:** ^1^Division of Neuroscience, Oregon National Primate Research Center, Oregon Health and Science UniversityBeaverton, OR, USA; ^2^Department of Behavioral Neuroscience, Oregon Health and Science UniversityPortland, OR, USA

**Keywords:** attentional set-shifting, non-human primates, cognitive flexibility, intradimensional discrimination, extradimensional discrimination

## Abstract

Attentional set-shifting ability is an executive function underling cognitive flexibility in humans and animals. In humans, this function is typically observed during a single experimental session where dimensions of playing cards are used to measure flexibility in the face of changing rules for reinforcement (i.e., the Wisconsin Card Sorting Test (WCST)). In laboratory animals, particularly non-human primates, variants of the WCST involve extensive training and testing on a series of dimensional discriminations, usually in social isolation. In the present study, a novel experimental approach was used to assess attentional set-shifting simultaneously in 12 rhesus monkeys. Specifically, monkeys living in individual cages but in the same room were trained at the same time each day in a set-shifting task in the same housing environment. As opposed to the previous studies, each daily session began with a simple single-dimension discrimination regardless of the animal’s performance on the previous session. A total of eight increasingly difficult, discriminations (sets) were possible in each daily 45 min session. Correct responses were reinforced under a second-order schedule of flavored food pellet delivery, and criteria for completing a set was 12 correct trials out of a running total of 15 trials. Monkeys progressed through the sets at their own pace and abilities. The results demonstrate that all 12 monkeys acquired the simple discrimination (the first set), but individual differences in the ability to progress through all eight sets were apparent. A performance index (PI) that encompassed progression through the sets, errors and session duration was calculated and used to rank each monkey’s performance in relation to each other. Overall, this version of a set-shifting task results in an efficient assessment of reliable differences in cognitive flexibility in a group of monkeys.

## Introduction

Cognitive flexibility is one of the essential executive functions underlying associative behaviors (Dajani and Uddin, 2015; Friedman and Miyake, 2017). Based on learning about negative or positive outcomes of an action in the presence of particular stimuli, decisions are made about further activity including altering strategies in response to changes in external rules or internal conditions (Izquierdo et al., 2017). Historically, cognitive flexibility was measured in humans and animals using variants of the Wisconsin Card Sorting Test (WCST; for review see Brown and Tait, 2016). Briefly, the WCST requires participants to sort cards based on dimensional qualities (e.g., color, number and suit). The rules for sorting the cards are sequentially altered and individuals are required to change their sorting strategy. A commonly used variant of the WCST is the Cambridge Neuropsychological Automated Test Battery (CANTAB) that requires a participant to repeatedly perform a discrimination of a set of stimuli, either of two simple stimuli (e.g., two lines) or two compound stimuli (e.g., shape/line combination). With compound discriminations, only one of the dimensions is the basis of the discrimination (e.g., the line of a line/shape combination). Normally the subject is given multiple trials of the same set of stimuli until a predetermined set of criteria is reached after which the subject can advance to a new discrimination set. An intradimensional discrimination (ID) shift in a set occurs when new exemplars (e.g., new lines/shapes) are presented but the same dimension (line) remains the basis of the discrimination (Dias et al., 1996b). An extradimensional discrimination (ED) shift occurs when new exemplars (e.g., new lines/shapes) are presented but the other dimension (shape) becomes the basis of the discrimination. Finally, following the acquisition of discrimination set, the relevant stimuli might be reversed such that previously incorrect choice stimulus of the set becomes the correct choice.

Both the WCST and CANTAB ID/ED tasks have been used for investigation of cognitive flexibility in non-human primates and adapted for experiments in rodents and other animals such as sheep (Crofts et al., 1999; Weed et al., 1999; Zürcher et al., 2010; Morton and Avanzo, 2011; Rodriguez et al., 2011; Horner et al., 2013). Usually, the experimental sessions take place in behavioral chambers remote from the housing environment. However, performance of non-human primates during these experiments may be improved when they are conducted in the animals’ housing cages, as demonstrated by Crofts et al. (1999) with marmosets and rhesus monkeys performing the CANTAB set-shifting task. Training and testing in the home-cage allows monkeys to stay in the same social and environmental contexts and, perhaps, observe and learn from performances of others (Myers, 1970; Subiaul et al., 2004; Meunier et al., 2007). Training in the housing environment also reduces training time and potential distraction involved in transferring monkeys to another environment, although remaining in the housing environment with visual, auditory and olfactory access to each other can also be a source of distraction. The ability to simultaneously assess cognitive function in a group of monkeys within their housing environment allows a comparison of individual performance without having a confound of individual response to transfer or testing at different times of the day. In addition to reducing between animal variability, simultaneous cognitive testing in the home environment greatly reduces technical effort in assessing large groups of primates. Finally, establishing simultaneous “housing-environment” measurement of set-shifting abilities more closely resembles the learning environment of a classroom compared to being sequestered alone in a sound and visually restrictive chamber.

The ID/ED discrimination tasks commonly used includes up to eight consecutive, one- or two-dimensional discriminations or sets, with each set presented as a series of trials in which the monkey chooses one of the two stimuli (or stages, for details Weed et al., 1999, 2008; Baxter and Gaffan, 2007; Nagahara et al., 2010). Normally, a correct choice is reinforced by the presentation of a flavored fluid or a small amount of food. Animals advance through the task by reaching a criterion for each discrimination set. The criteria for completion of discrimination are usually based on a preset number, ratio, or percentage of correct and/or incorrect trials. Using this procedure, most studies begin each daily training session with the discrimination set that was left without reaching criterion during previous session, presumably to maintain the task learning. Finally, number of trials or errors to criteria is analyzed and compared between subjects or groups (for example, Dias et al., 1997; Decamp and Schneider, 2004).

The present study was designed to provide monkeys with opportunity to reach criteria for all eight discrimination sets within a session. Moreover, we utilized a second order reinforcement schedule to reduce the probability of satiation and increase the probability that monkeys will continue to perform during the 45 min session. The study aimed to determine if the cognitive flexibility of individual monkeys could be assessed both within- and between-subjects based on their relative performance. Briefly, a custom set-shifting procedure was developed that included eight different discrimination sets displayed onto a computer controlled touch screen integrated into the side of each housing cage. The computer program randomized and displayed the visual sets side by side, acquired the choice data and provided visual feedback for correct or incorrect choices. Each daily session began for all monkeys at the same time with the first (simple) discrimination set, and monkeys progressed through the discrimination sets according to their own pace and abilities. We hypothesized that the performance of monkeys on the set-shifting task would gradually improve over the daily sessions, demonstrated by a decrease in the number of errors per session and by an increase in the number of sessions when all eight discriminations sets were completed to criteria. We also hypothesized that reversal discriminations would be more difficult, i.e., result in a larger number of errors than the original discrimination.

## Materials and Methods

### Animals

Twelve late adolescent/young adult rhesus monkeys (*Macaca mulatta*) weighing 5–6 kg and approximately 4.5 years at the beginning of the study were used. Animals were born in the breeding colony of Oregon National Primate Research Center and weaned at about 2 years of age and placed in same sex peer groups with a few adults. The monkeys were experimentally naïve and upon assigned they were housed indoors in a room with metal housing cages (0.8 × 0.8 × 0.9 m) capable of vertical partition removal to create pair housing. The housing room allowed the caging to be arranged in two rows facing each other and had controlled temperature (20–22°C), humidity (65%), and an 11-h light cycle with lights on at 07:00 AM. Food, in the form of nutritionally complete 1 g banana-flavored pellets (TestDiet, St. Louis, MO, USA) was provided in one meal 2 h after the set-shifting sessions each day. Water was available* ad libitum*. Animals were housed in pairs 1–2 h between 9 AM and 11 AM daily. All procedures in this study were conducted according to the Guide for the Care and Use of Laboratory Animals and approved by the Oregon National Primate Research Center Institutional Animal Care and Use Committee.

### Apparatus

Each monkey cage was equipped with a modified version of the operant panel previously described (Grant et al., 2008). For this study, the panel was modified to incorporate a LCD 11 × 13.25″ monitor (Dell Inc., Model E1715S) with touch-sensitive screen (Keytec, Inc., Model OPTIR Touch PPMT) attached to the monitors. The touch screen and all inputs and outputs were controlled by a computer system using custom-made software (LabView 2011, SP1, National Instruments).

### Picture Preference Test

All animals were trained to use the touch screen with a picture preference test prior to the set-shifting training. Monkeys were given nine sessions conducted once per day. Each session began by displaying a pair of photographic images (250 px, 8.2 cm) side-by-side for 15 s. If the monkey touched one of the images within 15 s, that image was enlarged to a full-screen display (1024 mm × 768 mm) for 30 s followed by 15 s inter-trial interval. If no image was touched in 15 s, there was a 15 s interval before the same combination of images was displayed. The photographic images for the test were taken at the ONPRC and grouped into four categories: nature (images of scenery, no animals or humans), laboratory (pictures of lab personnel familiar to the monkeys), monkey affiliative (pictures of rhesus monkeys together, grooming or nurturing) and fruit (images of fruit). Each category was matched to a different category, creating six unique category pairings. Then, six images within each category were displayed alongside each of six unique pictures from the matched category. Thus six category matchings of 6 unique pictures resulted in 36 trials. During sessions 7–9, two additional categories were added: aggression (pictures of rhesus monkeys threatening or aggressively postured) and cynomolgus (pictures of cynomolgus monkeys in home cages). This increased the number of the category pairings to nine and trials to 54. Importantly, no training was required for the monkey to touch the images; they did this spontaneously. The preferred pictures were used in the set-shifting paradigm and signaled correct responses. It should be noted that the number of correct trials did not need to be consecutive.

### Set-Shifting Paradigm and Training

**Figure 1:**
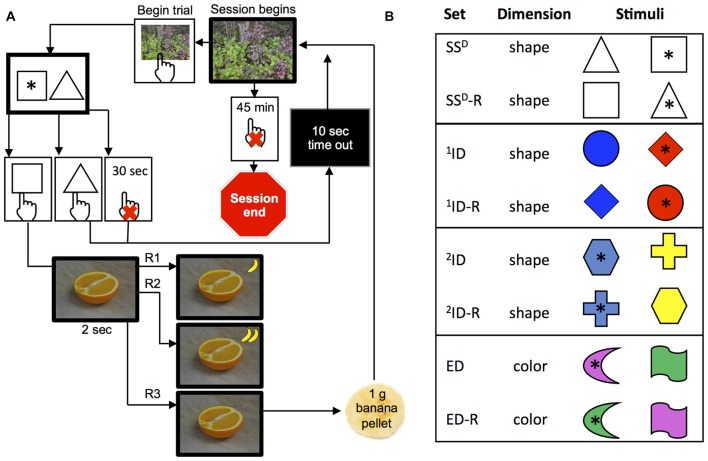
**Set-shifting paradigm with the second-order FR3 schedule of reinforcement. (A)** Schematic representation of a trial. Scenery photograph was presented to indicate initiation of trial. The monkey had to touch the photograph to begin a trial, which was the presentation of two distinct shapes: black or white in sets 1–2 (simple discrimination stimuli) and colored in sets 3–8 (compound discrimination stimuli). The stimuli were presented for 30 s before the trial timed out and this was recorded as an incomplete trial. If the monkey touched a correct shape * within 30 s, then a photograph from the “preferred” category (e.g., an orange) and a small icon of a banana were displayed for 2 s. If the monkey touched an incorrect shape or did not touch any shape within 30 s, then the screen went dark and a 10 s time out period was initiated. **(B)** Schematic representation of four discriminations and reversals. Relevant dimension for each set is indicated in the second column: shape or color. Session ended after 45 min or if the monkey reached criteria (12/15) in all eight sets.

The set-shifting paradigm used a battery of eight stimulus-discrimination levels with increasing complexity (four discrimination “sets” and four reversals of these sets), which could be completed within a single behavioral session (Figure 1). Sessions were conducted between 11 AM and noon (4 h after the room lights were on), 6–7 days a week, during a 5-week period (34–37 session total). The number of sessions varied for individual monkeys due to other scheduled procedures (MRI, physical exams, etc.). Each session was limited to 45 min (session time), but could end sooner if criteria for acquiring all discrimination sets were met. Number of trails was limited by the session time, individual rates of responding as well as incomplete or incorrect trials resulting in a time-out (see below). The criterion for acquiring discrimination was a running total of 12 correct trials out of 15 consecutive trials. The rate at which the monkey advanced through the session was self-paced and depended on the successful acquisition of each of eight discriminations.

Each trial within a discrimination set began with a presentation of full-screen neutral photograph from a stock of six nature photos (i.e., trees, sky, pond, etc., Figure 1A). The monkey had to touch the neutral photograph in order to initiate the trial and this same image was presented to start each trial of the session, across all discrimination sets. If the monkey did not touch the neutral photograph within 45 min, the session was programed to end (this never occurred). When the monkey touched the neutral photograph, two discrimination stimuli (8.2 cm × 8.2 cm) appear side-by-side on the screen. The shape and color of stimuli presented depended on the discrimination set. At each discrimination set, one of the stimuli was randomly designated as correct on the first trial and remained as the correct discrimination “rule” for each trial until criteria for discrimination was met (12 correct trials out of 15 consecutive trials). The monkey had to touch one of the stimuli within limited hold of 30 s.

In order to keep all monkeys engaged in the task for the 45 min sessions or until all eight sets were completed, we implemented second schedule of reinforcement as a novel methodological variation of the following set-shifting program. For the first 2–4 sessions, the monkeys were on a fixed ratio schedule of 1 (FR1), where touching the correct stimulus within the limited hold immediately changed the screen from the discrimination choice to a preferred photograph from one of three categories (fruit, lab personnel, or affiliative; see above) for 2 s and a 1 g banana pellet was delivered. Monkeys advanced to a FR second order schedule of pellet delivery (maintaining a FR1:picture schedule) if they had less than 10% of their trials ending due to an incomplete trial (initiating a trial but not touching a stimulus within a limited hold of 30 s). Thus, after 2–4 sessions, a second order FR2 schedule of pellet delivery for correct trials (maintaining a FR1:picture schedule) was implemented, in which the first correct response resulted in the presentation of preferred photograph and a small banana icon (2.1 cm diagonal) in the upper right-hand corner of the screen, and then the second correct response (trial) resulted in the preferred photograph, a second banana icon, and a banana pellet. Upon delivery of the pellet, the banana icons at the top right of the screen were removed and the next trial began. Finally, after a maximum of 10 sessions for all monkeys with exception of two animals (#581 and #423; 25–26 sessions), the schedule was increased to FR3 (Figure 1A) of pellet delivery where a pellet was delivered after the third correct trial, and the preferred photographs continued to be presented after every correct response (FR1:picture schedule). Importantly, the correct trials did not need to be consecutively for the FR3 schedule of pellet delivery nor for the FR1 schedule of picture delivery. The banana icon was a visual display of the correct trials toward FR completion from trial to trial. Touching an incorrect stimulus or not touching any stimulus within limited hold of 30 s changed the screen from the discrimination choice to black for 10-s timeout.

Monkeys began every set-shifting session with the simple discrimination set, where the discrimination of two objects presented side-by-side is based on the shape of the black or white object (for example, a white triangle and a white square, Figure 1A). During each discrimination set, monkeys had to learn which stimulus is correct based on “hit or miss process”. After the criterion of a running total of 12 correct out of 15 trials was reached, either a reversal of the correct stimulus for the current set or the next discrimination set began. Figure 1B represents the bases of the discrimination for each of the eight sets that could be acquired in a single session. The first discrimination level was a simple stimuli-discrimination (SS^D^) and was based only on shape (no colors present). The second discrimination level was the simple discrimination reversal (SS^D^-R) of the same two shapes, where the opposite shape was correct but all other aspects of the discrimination remained consistent. The third discrimination level presented compound stimuli (shape and color), with the colors of the shape changing randomly between two shapes. In this ID, the monkey had to learn the new shape that was the basis of the discrimination but ignore color. The fourth discrimination level (^1^ID-R) was a reversal where the opposite shape was correct, but all other aspects of the discrimination remain. The fifth discrimination (^2^ID) was similar to the first ID; the monkey was presented with new compound stimuli (two new shapes and two new colors different from previous sets). The sixth discrimination level was a reversal (^2^ID-R), such that the same two shapes and two colors as in the previous discrimination were randomly presented, but the opposite shape was correct. Thus two IDs and reversals were used in this study. The seventh discrimination (ED) was an ED, where two new shape and two new color combinations were chosen, with the colors of the shape changing randomly. However, *color* was the correct basis of the discrimination and thus this was an ED (from shape to color). The eighth and final, discrimination level (ED-R), randomly presented the same two shapes and two colors on the two sides, but the opposite color of the seventh discrimination was correct. Excluding reversal discriminations, the shape or color was only used once and chosen randomly (without replacement) from the shapes listed in Figure 1B. Location of the correct stimuli on the screen was varied side to side from trial-to-trial across all eight discrimination levels.

### Data Analysis

The dependent variables used for analyses were: the total number of trials/session, the number of correct trials/session and errors/session, the number of incomplete trials/sessions, the number of sessions to criterion performance within each set, the maximal set reached at the end of a session, the session duration and the ratio of number of errors/number of trials per session. Two-way repeated measures analysis of variance (ANOVA) was used with number of trials to criterion as a variable, trials outcome (correct and errors) as a between-subject factor and sessions as within-subject factor. Data from the ANOVA were considered significant if *p* value was less than 0.05. In order to assess individual differences across monkeys, a performance index (PI) was calculated for each animal based of three factors: the set that a monkey reached at the end of a session (“set”), the session duration (“duration”) and the ratio of number of errors in the session to trials (“errors”). The raw data for each factor in each session were normalized by scaling from 0 (worst) to 100 (best) using the equations (1–3):

(1) $$a\;=\;\frac{x-1}{8-1}*100,$$

where *a* indicates the factor “set”, *x* is the maximal set animal reached during a session, 1 and 8 are minimal and maximal set an animal could possibly reach. Thus, if an animal reached all eight sets during a session, his performance would be scored as 100 on this factor.

(2) $$b\;=\;\frac{x-2700}{192-2700}\;\text{*}\;100,$$

where *b* indicates the factor “duration”, *x* is the duration of a session, 2700 and 192 are maximal and minimal durations. The maximal session duration was defined by the preset session time (in s) of 45 min. The minimal session duration was defined by adding the time (in s) it would require if every response was correct for every trial in every set. Specifically, the duration of the preferred picture (photograph) display after a correct response was set to 2 s Thus, 2 s multiplied by 12 (the minimal trials to criteria) multiplied by 8 (sets) = 192 s. Thus, based on formula (2) if an animal used 2700 s to complete a session, his performance would be scored as 0 on this factor.

(3) $$c\;=\;\frac{x-1}{0-1}*100,$$

where *c* indicates the factor “errors”, *x* is the ratio of number of errors per session to number of trials per session. 1 and 0 are the maximal and minimal possible ratio. Similar to factor “duration”, the ratio 1 indicates the poorest outcome on the task and all trials ended in an error during a session. Thus, based on formula (3) if an animal had ratio of number of errors to number of trials equal to 1, his performance would be scored as 0 on this factor.

After the scaling, the PI of each session was calculated for each monkey using the equation (4):

(4) PI = a+b+c,

where *a, b* and* c* are scaled data for three factors averaged across the final 10 sessions for each monkey. Thus, the maximal possible index of performance could be 300.

## Results

**Figure 2:**
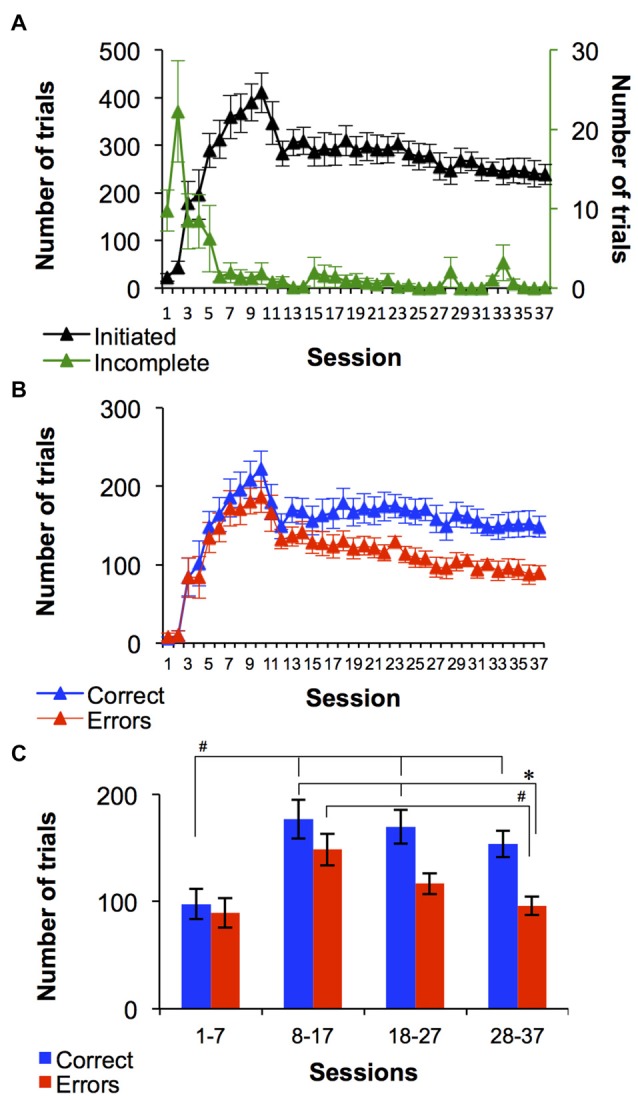
**Average set-shifting performance of the 12 monkeys. (A)** Average number of initiated and incomplete trails over 37 sessions. **(B)** Average number of correct trials and errors. **(C)** Average number of correct trials and errors collapsed into four bins, where 1st bin includes the seven initial sessions and the next three bins are the remaining 30 sessions divided into 10 session bins. All graphs demonstrate task-related performance of monkeys regardless set reached during the session. Data are mean ± SEM. * and ^**#**^Indicate significant between-group and within-group differences, respectively (Bonferroni test all *p* < 0.05).

An important property of the procedure designed in this study was that monkeys initiated each trial during every session by touching a photograph displayed on the screen. Average number of trials initiated across the entire group *regardless of the discrimination set* is shown in Figure 2A. The number of trials increased robustly from session 1 (23 ± 8 trials) to session 10 (410 ± 42 trials). During sessions 11 and 12 the number of trials decreased to 283 ± 27 and by the end of training the average number of trials/monkey was 238 ± 21. In the first two sessions the monkeys typically initiated trials but did not touch a shape for a group average of 9 incomplete trials out of 23 total trials during session 1 and 23 incomplete trials out of 43 during session 2 (Figure 2A). However, by the third session the average number of incomplete trials/session in this group of 12 monkeys decreased to 11 ± 4 out of 178 ± 46 total trails/session. Notice that during initial period of the training (session 1–10), the second order FR schedule of pellet delivery for correct trials progressed from FR1 to FR3. Eight out of twelve monkeys (67%) progressed to FR3 by session 7. The change in the second order FR schedule contributed to the observed increase in the number of trials (especially during sessions 7–10), which correlated with the increase in number of correct responses (Figure 2B).

Figure 2B shows the average number of correct trials and errors that occurred in each session, across all monkeys. On average, the performance on the first two sessions reflected random behavior. Specifically, during session 1 there was an average of 6 ± 3 correct trials and 8 ± 5 errors. In the second session there was an equal number of correct trials and errors (10 ± 6). During sessions 4–16, the average number of correct trials was similar to the average number of errors (Figure 2B) and the number of incomplete trials decreased to near 0 (Figure 2A). Across sessions 17–37 the number of errors declined while number of correct choices remained relatively consistent. There was main effect of trial outcome (*F*_(1,8)_ = 13.6, *p* < 0.01) and time (sessions; *F*_(36,288)_ = 21.8, *p* < 0.0001), with an interaction between the factors (*F*_(36,288)_ = 1.5, *p* < 0.05). In Figure 2C, the number of correct trials and errors are given for the first 7 sessions as a single bin and subsequent 30 sessions are shown as 10 session bins (Figure 2C). The number of errors decreased over the last 30 sessions, and the difference between correct trials and errors became greater. A two-way repeated measures ANOVA on the collapsed data revealed a main effect of trial outcome (*F*_(1,22)_ = 4.8, *p* < 0.05) and time (sessions; *F*_(3,66)_ = 26.2, *p* < 0.0001), with a significant interaction between the factors (*F*_(3,66)_ = 4, *p* < 0.05). Bonferroni *t*-test yielded a significant difference between the number of errors during sessions 28–37 and the number of correct trials during sessions 8–17 and 18–27 (all *p* < 0.05), as well as number of errors during sessions 8–17 (*p* < 0.001). Additionally, Number of correct trials was significantly higher during sessions 8–17, 18–27 and 28–37 compare to sessions 1–7 (Bonferroni *t*-test, *p* < 0.05).

**Figure 3:**
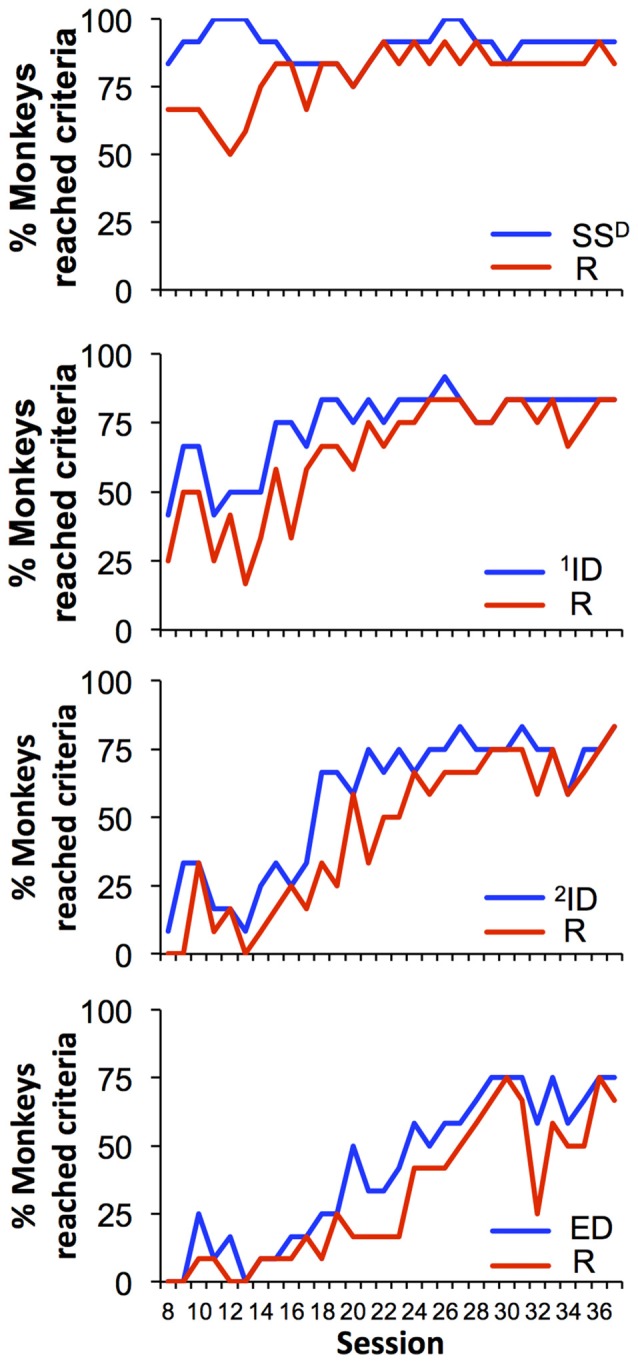
**Percent of monkeys (out of 12 total) reaching criteria for each set of the task.** Each graph demonstrates data for two sets: original discrimination set (blue) and its reversal (red).

Every session began with the simple discrimination set and the possibility that each monkey could reach the criteria for all eight sets within a single 45-min session. Figure 3 demonstrates the percent of monkeys that reached the criterion of 12/15 correct trials for each of eight discrimination levels across the final 30 sessions (from 8 to 37). During session 8, 83% of the monkeys (10/12) reached the criterion for the simple discrimination and 67% of monkeys (8/12) reached criterion for the simple discrimination reversal. This high level of performance across this cohort of animals was observed during the remainder of the sessions. The percentage of animals that reached criteria for subsequent sets and reversals generally decreased with increasing complexity of the discriminations. For example, in session 8, only 42% and 25% of monkeys reached criterion for ^1^ID and its reversal, respectively, whereas only one monkey reached criterion for ^2^ID. Further, in session 8 no monkey reached criteria for any discrimination at set 7 and beyond. However, performance on the task improved across the consecutive sessions. By the end of the experiment (session 37), 83% of animals were capable of reaching criteria for both the intradimensional sets and their reversals and 75% of monkeys reached criteria for extradimensional set and 67% for its reversal.

**Figure 4:**
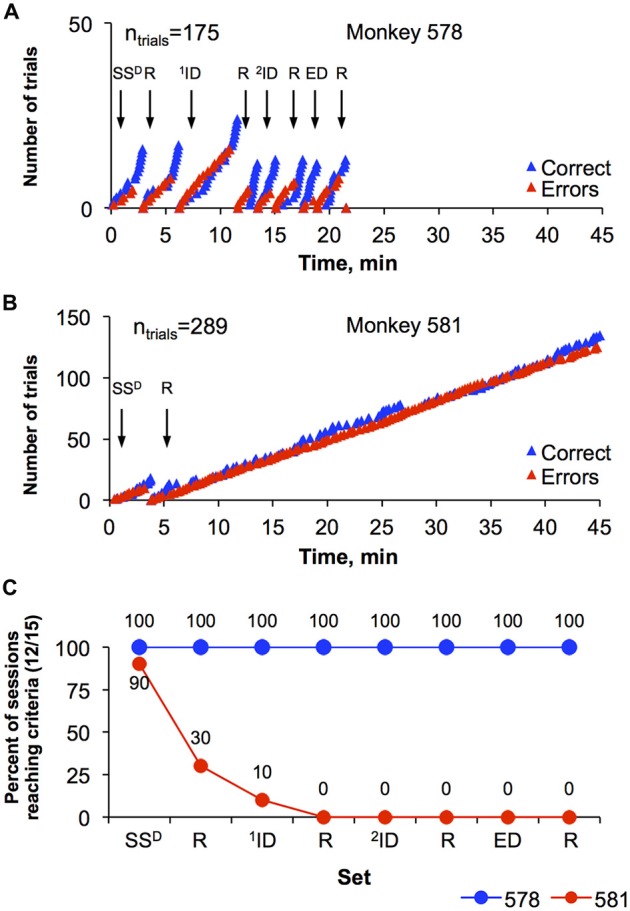
**Representation of set-shifting performance by two monkeys. (A)** Cumulative response records during the last set-shifting session showing the number of correct and incorrect trials conducted by monkey 578 in session 37. Arrows with set-specific acronyms above them indicate initiation of a new set within the session. Advancing to a new set required 12 correct trials (blue triangles) out of 15 consecutive trials. Once the set was completed, a new set began. The total number of trials (*n*_trials_) for this session is shown in the top left corner of the graph. **(B)** The number of correct and incorrect trials conducted by monkey 581 in session 37. Details are the same as graph in **(A)**. **(C)** Overall performance on the set-shifting paradigm by monkey 578 and 581 during 10 last sessions (28–37). Data are in percentage of sessions when a certain set was completed. The acronyms are: S^D^, stimulus discrimination; R, reversal for any previous set; SS^D^, simple S^D^; ID, intradimensional discrimination; ED, extradimensional discrimination.

One of the major goals of this study was to design a set-shifting procedure that allows comparison of cognitive flexibility of individual monkeys. Figure 4 represents performance of two monkeys (578 and 581) during session 37, the final session. The monkeys represent one of the top ranked monkey (best) and the bottom ranked monkey (worst) performances using this dimension of the task. Specifically, monkey 578 engaged in total of 175 trials and completed all eight sets of the session in 21.4 min (Figure 4A). In contrast, monkey 581 engaged in 289 trials, completed first set (SS^D^) in 3.3 min but did not reach performance criteria in the reversal (SS^D^-R) before the session ended at 45 min (Figure 4B). Figure 4C is a depiction of overall performance of the monkeys (578 and 581) and their progression through the eight levels of the set-shifting during final 10 sessions (sessions 28–37, when the number of errors decreased). Thus, Figure 4C shows the percentage of the 10 sessions where criterion (12 of 15 correct trails) was reached for each the eight levels of the set-shifting task. Monkey 578 completed all eight sets during 100% of the sessions. In contrast, monkey 581 was less successful and completed S^D^ in 90%, S^D^-R in 30%, ^1^ID in 10%, and never reached ^1^ID-R, ^2^ID, ED and their reversals.

**Figure 5:**
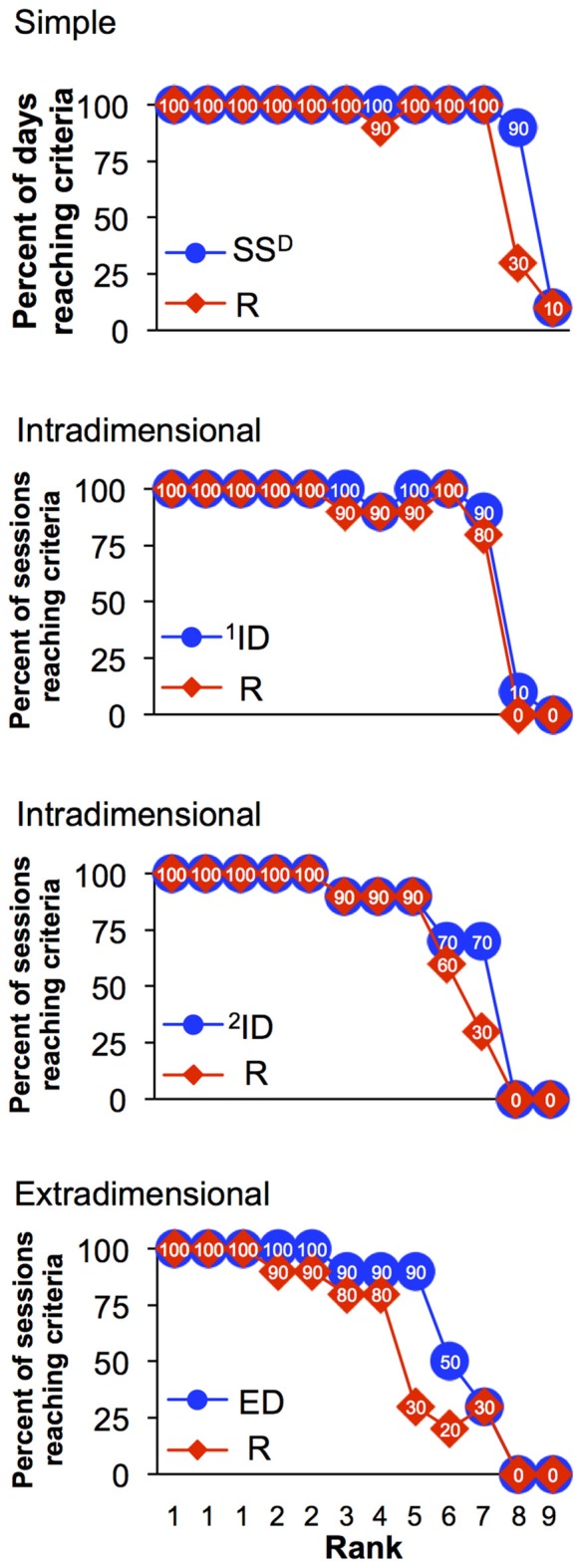
**Rank order of monkeys based on their performances on the set-shifting task during last 10 sessions (sessions 28–37), separated by the discrimination level and its reversals (i.e., simple discrimination, two intradimensional (ID) and one extradimensional discrimination (ED)).** Each monkey was given a rank order according to the percentage of sessions when it reached certain discrimination set.

It is possible to use the analysis shown in Figure 4C to rank the performance of the 12 monkeys as shown in Figure 5 giving the rank order of 12 monkeys in completing all levels of the set-shifting task in the final 10 sessions. However, using the single factor of percentage of sessions when an animal completed each discrimination level, it is not possible to distinguish between the top three monkeys, which were tied with 100% of session reaching set 8 and thus shared a Rank of 1. Overall, Figure 5 demonstrates that percentage of sessions when animals reached higher discrimination sets decreases with the increasing complexity of the sets and especially with performance of reversal sets. However, this single factor is insufficient to clearly assign individual ranks to each of the 12 subjects.

**Figure 6:**
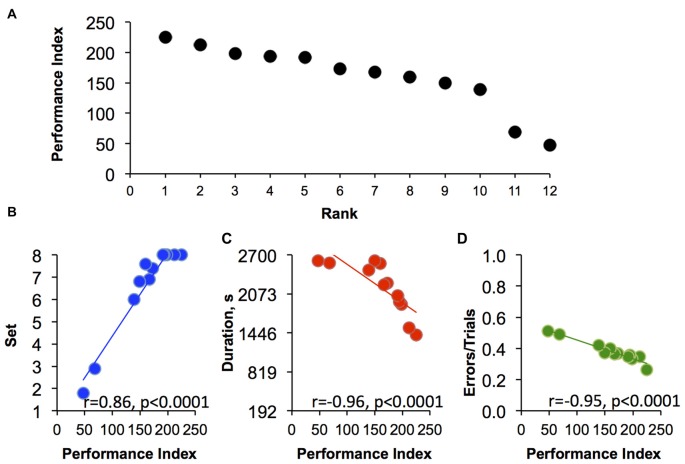
**The three-factors rank order. (A)** The correlation between index of performance and three-factors rank orders (Rank). **(B–D)** Correlations of performance index (PI) in relation to the three factors (“set”, “duration” and “errors”; see “Materials and Methods” Section for more details) that comprise the PI (*n* = 12). Each point is the average of last 10 sessions for **(B)** set reached at the end of the session, **(C)** session duration and **(D)** the ratio of errors to total number of trials per session. All correlations were analyzed using Spearman’s rank-order test.

To overcome similar performance rank using a single factor as in Figure 5 (% sessions attained in each discrimination set), we extended our analysis by combining three factors that appear to each contribute to overall performance in set-shifting abilities (Figure 6). As shown in Figure 6A, all 12 monkeys could be distinguished from each other and ranked ordered from 1 to 12 using the three-factor analysis. These factors include the set the monkey had reached by the end of a session, the session duration and the total number of errors/total session trials. Each of these factors were normalized on a scale of 0%–100% and were mathematically added to calculate a single index of performance (see details in “Materials and Methods” Section). Individual index of performance in this set of monkeys ranged from 47 to 225, where smallest index represents poorest performance on the task. Figure 6 shows the strong and significant correlation of each factor (“set”, “duration” and “errors”) with the index of performance. Thus, animals with high indexes of performance on this set-shifting procedure tended to reach all eight sets of the task within short period of time and with small number of errors during the sessions.

## Discussion

In this study we developed a unique set-shifting procedure for efficient and individualized assessment of cognitive flexibility in a group of rhesus monkeys. Twelve monkeys were simultaneously trained and tested in their “housing” environment where they had visual, auditory and olfactory access to each other. The daily set-shifting sessions were integrated into the monkeys’ daily routines like meals, social interactions, sample collection, cleaning, etc. Previous studies have tested cognitive functions of individual monkeys within the home environment (e.g., Gazes et al., 2013; Tu et al., 2015). For example, Gazes et al. (2013) assessed rhesus monkeys that were pair-housed in a laboratory setting, and separated for testing using a touch screen attached to the housing cage during the testing procedure. The performance on a battery of perceptual and cognitive tasks of the laboratory monkeys were compared to monkeys housed in a social group within a field station environment equipped with 4 touch screen stations (Gazes et al., 2013). In addition, Crofts et al. (1999) demonstrated the ability of monkeys to perform on a set-shifting ID/ED test where individual monkeys were tested in a designated compartment of the home-cage. However, for a majority of studies investigating cognitive performance in monkeys, the training and testing usually occurs in a separate room, testing cage, or restraint chair away from the housing environment (Weed et al., 1999; Decamp and Schneider, 2004; Zürcher et al., 2010; Kromrey et al., 2015). Finally, in all the studies cited above, only group data were presented and individual differences were not analyzed. In the procedure presented here, the data could be subjected to group or individual analyses.

Thus, unique to this study was the incorporation of the touch-screens into the housing cages, the simultaneous implementation of the set-shifting task to all monkeys, and the start of each session with the lowest level of discrimination regardless of their previous session performance. These modifications to previous assessments of set-shifting abilities in monkeys resulted in a robust method of training, testing and rank ordering animals based on an aspect of cognitive flexibility (Brown and Tait, 2016). The steep increase of number of completed trials over first 10 sessions (Figure 2A) show that monkeys learned to initiate the trials very quickly and to respond under a second order FR1–3 schedule of pellet delivery for correct trials. After this initial period, the group data show the number of correct stimulus choices remained consistent across the sessions and the number of errors decreased indicating that, as a group, the monkeys were learning to anticipate reversals and multidimensional shifts in the discrimination tasks. Importantly, this procedure provides nearly identical training history for all subjects and is applicable for longitudinal analysis of flexible cognitive behavior at the level of an individual animal, but behaving within group setting.

A major difference between studies of cognitive function in humans and animals is the long-term training in laboratory animals. While the cognitive testing in humans usually occurs during a single day, in animals it is a long-term process until preset performance criteria are reached and performance is evaluated (Decamp and Schneider, 2004; Weed et al., 2008; Zürcher et al., 2010; Rodriguez et al., 2011). Thus, the number of sessions required to learn a single discrimination across individual monkeys is highly variable, and few studies report the number of training sessions needed to establish the consecutive discriminations in traditional set-shifting tasks. The present procedure used a different approach of all animals receiving the same number of sessions and equal opportunities to progress across the sets without any limit on number of trials they initiate. Another difference between the current procedure and previous studies in monkeys is that here, every set-shifting session began with the first, simple discrimination set. This ensured that, all monkeys were subjected to the same discrimination stimuli as their performance advanced across sets within a session, making comparisons across monkeys less confounded on this dimension. In contrast, many of the previous assessments using set-shifting began each session with the discrimination that was reached during the previous session in order to advance to more difficult discriminations, but giving subjects differential experience with across session stimuli. Finally, in this procedure the entire number of sessions from onset to a complete data set required about 5 weeks (34–37 sessions), with 10 out 12 monkeys reliably performing under the terminal second order schedule within eight sessions (Figure 3). Importantly, more than 83% (10 out of 12 monkeys) of animals reached criterion for reversal of the extra-dimensional discrimination (the 8th level) by the end of the 5 weeks.

This procedure also provided evidence that each subsequent discrimination set (sets 1, 3, 5, and 7) are more difficult to acquire and the reversals of these discriminations (sets 2, 4, 6, 8) are more difficult that the original acquisition sets. This result is demonstrated by the decrease in the number of sessions that met the criteria for advanced sets across the group (Figure 3). Specifically, the simple discrimination (SS^D^) appeared to be the easiest set to learn, as most of the monkeys (10 out 12) completed it nearly every session during the last 10 sessions. A similar trend was observed for the reversal of the SS^D^ (SS^D^-R). This level of difficulty corresponds to previous studies where the fewest errors to reach criteria were recorded during simple discrimination compared to reversal sets (for example, Weed et al., 2008; Zürcher et al., 2010). Likewise, the percentage of sessions reaching the criterion decreased with the increase in the complexity discrimination from simple to ID to ED. The final set of ED discriminations was only completed on average in 70% of the last 10 sessions, and the individual differences are striking, with the top ranked monkey reaching the criterion for ED in 100% of the sessions, compared to four lowest ranked monkeys that reached criterion for ED in less than 50% of the sessions (Figure 5).

The data analyses explored how best to rank order cognitive performance across the 12 monkeys studied. Using only within session progress in advancing through the increasingly difficult discrimination sets (Figure 3), the two monkeys with the lowest ranks were quite distinguishable among the other monkeys based on the task performance (Figure 5). Importantly, the behavior of the lowest ranked monkeys illustrated that the failure to advance to the next, more difficult level was not due to being unengaged in the task, but rather due to a failure to acquire the simplest discrimination in a majority of sessions despite completing hundreds of trials (Figure 4B). Using only a single factor (i.e., set reached) was not adequate to differentiate all monkeys and the top three monkeys shared the same rank (Figure 5). Using a 3-factor index of performance (Figure 6), where behavior during the session was based on the set that monkeys were able to reach during the sessions, plus the normalized duration of each session and a normalized error per trial value complete separation of rank order was possible. There was a strong correlation for each of the three factors with the PI across individuals, with top ranked monkeys completing their sessions earlier then preset session time of 45 min, reaching all eight discriminations, and showing a high level of accuracy (small number of errors per total number of trials). For example, the two monkeys with the poorest performance responded incorrectly in 50% of trials (Figure 6C) and there was a clustering of individuals for the factor “error”, which suggest that using only this factor is not sufficient for rank ordering the performance of the monkeys. The addition of the session time added a necessary complement to both errors/trials and set reached. Specifically, the monkey with highest index of performance completed the sessions in an average of 1415 s after start, which is only 52% of the allowed time per session. Because most studies consider only the number of trials or number of errors to criteria as a single variable for analysis of animal’s responding on the task (Weed et al., 2008; Maeda et al., 2014; Freeman et al., 2015), it does not allow the speed at which a monkey is able to learn multiple sequential discriminations to be evaluated. However, this may be an important aspect of primate engagement in cognitive tasks. Indeed, the highest ranked monkeys were actually obtaining less than half the banana pellets than the lowest ranked monkeys in the final 10 sessions. The motivation for advancing through more difficult cognitive tasks cannot be explained by the explicit schedule of reinforcement and suggests increasingly difficult cognitive tasks may have inherent reinforcing value to some primates.

Cognitive testing in monkeys is used to model a variety of human psychiatric disorders associated with alteration of cognitive flexibility and behavioral disinhibition. Set-shifting tasks can be used to explore discrete brain structures and functional networks that are potentially involved in the control over the behavioral flexibility (Morris et al., 2016). While this study does not explore brain mechanisms supporting the set-shifting abilities in monkeys, the previous experiments combining set-shifting tasks and brain lesions in nonhuman primates revealed the predominance of lateral prefrontal cortex in the control over attentional focus during the intradimensional and extradimensional shifts and the orbitofrontal control over reversals (Dias et al., 1996a). In humans, similar cortical control over the set-shifting functions is accompanied by activation of the posterior parietal cortex (Hampshire and Owen, 2006). Importantly, besides the cortical input, a number of subcortical structures contribute to the regulation of the cognitive flexibility, for example, the caudate nucleus, amygdala and hippocampus (Graham et al., 2009). There is no doubt that individual differences in the development and functioning of these brain areas greatly contribute to the behavioral performance on the set-shifting procedures in human and animal subjects. Therefore, an important element in set-shifting testing is to identify individual differences in acquisition and performance. Allowing the individual to advance to increasingly more difficult learning sets at their own pace while in a group setting has some analogous aspects with a classroom.

There are several limitations to the current evaluation of set-shifting in monkeys. Primary among these is the group setting approach in which all monkeys are given the same task, at the same time. Although this is a very efficient procedure for evaluating a large number of monkeys, each individual’s performance on cognitive tasks in a group environment might be differentially affected by social factors or distraction by the behavior of group-mates (Snyder K. et al., 2015; Snyder K. P. et al., 2015). Although this may be an advantage for evaluating attentional deficits in social settings, it confounds interpretation of what factors are influencing learning. A second limitation is that there was no attempt to prolong training so that every monkey could reach the same criteria on each level of the set-shifting task. Prolonged training (or overtraining) to minimize between subject variance has been used to investigate the effect of manipulations to improve or impair performance. In the present procedure, individual differences would first have to be normalized across individuals (i.e., percentage of average baseline data) if this approach were to be taken. Finally, this approach confounds advancing to more difficult discrimination sets (i.e., simple to compound intradimensional to extradimensional sets) with the ability to reverse the contingencies of an acquired “rule”. That is, individuals that perform poorly on reversal were not allowed to (but may have been capable of) acquire the next set. Since reversal learning involves distinct cortical mechanisms from discrimination acquisitions (Dias et al., 1996a), imposing reversals after the acquisition of every set may obscure the differential ability to acquire new cognitive sets. Characterizing performance on only set acquisition without reversals could disentangle this confound if brain circuitry is specifically being investigated for mechanistic understanding of performance in a group setting.

In summary, we found the set-shifting abilities of monkeys performing in a group setting could be trained efficiently and yield a robust characterization of individual cognitive flexibility. As hypothesized, the performance of monkeys on the set-shifting task gradually improved over the course of study but individual differences in this measure of cognitive flexibility were easily measurable and apparently stable. The results suggest that this procedure can be used to capture cognitive performance in relatively short time frames for designs testing temporally distinct phases, such as stages of development, drug treatments, short term response to stressors, etc. The results also demonstrate that the rhesus monkey does not need to be physically isolated in order to learn and become stable on cognitively demanding tasks, allowing for use of the “classroom” model to assess learning and performance.

## Author Contributions

TAS analyzed the experimental data and prepared the manuscript. DCA prepared the experimental data and gave comments on the manuscript. NARW gave comments on the manuscript. SWG prepared experimental data and gave comments on the manuscript. KAG designed the study, analyzed the experimental data and prepared the manuscript.

## Funding

This study was funded by the National Institute on Alcohol Abuse and Alcoholism U01 AA013510 (to KAG), R24 AA019431 (to KAG) and Grant # P60 AA10760 (NIH).

## Conflict of Interest Statement

The authors declare that the research was conducted in the absence of any commercial or financial relationships that could be construed as a potential conflict of interest.
